# Identification of Tsetse (*Glossina* spp.) Using Matrix-Assisted Laser Desorption/Ionisation Time of Flight Mass Spectrometry

**DOI:** 10.1371/journal.pntd.0002305

**Published:** 2013-07-11

**Authors:** Antje Hoppenheit, Jayaseelan Murugaiyan, Burkhard Bauer, Stephan Steuber, Peter-Henning Clausen, Uwe Roesler

**Affiliations:** 1 Freie Universität Berlin, Zentrum für Infektionsmedizin, Institute for Parasitology and Tropical Veterinary Medicine, Berlin, Germany; 2 Freie Universität Berlin, Zentrum für Infektionsmedizin, Institute of Animal Health and Environmental Hygiene, Berlin, Germany; 3 Federal Office of Consumer Protection and Food Safety (BVL), Berlin, Germany; Johns Hopkins Bloomberg School of Public Health, United States of America

## Abstract

*Glossina (G.)* spp. (Diptera: Glossinidae), known as tsetse flies, are vectors of African trypanosomes that cause sleeping sickness in humans and nagana in domestic livestock. Knowledge on tsetse distribution and accurate species identification help identify potential vector intervention sites. Morphological species identification of tsetse is challenging and sometimes not accurate. The matrix-assisted laser desorption/ionisation time of flight mass spectrometry (MALDI TOF MS) technique, already standardised for microbial identification, could become a standard method for tsetse fly diagnostics. Therefore, a unique spectra reference database was created for five lab-reared species of riverine-, savannah- and forest- type tsetse flies and incorporated with the commercial Biotyper 3.0 database. The standard formic acid/acetonitrile extraction of male and female whole insects and their body parts (head, thorax, abdomen, wings and legs) was used to obtain the flies' proteins. The computed composite correlation index and cluster analysis revealed the suitability of any tsetse body part for a rapid taxonomical identification. Phyloproteomic analysis revealed that the peak patterns of *G. brevipalpis* differed greatly from the other tsetse. This outcome was comparable to previous theories that they might be considered as a sister group to other tsetse spp. Freshly extracted samples were found to be matched at the species level. However, sex differentiation proved to be less reliable. Similarly processed samples of the common house fly *Musca dome*stica (Diptera: Muscidae; strain: Lei) did not yield any match with the tsetse reference database. The inclusion of additional strains of morphologically defined wild caught flies of known origin and the availability of large-scale mass spectrometry data could facilitate rapid tsetse species identification in the future.

## Author Summary

Tsetse flies are confined to tropical Africa and are carriers for trypanosomes, single-celled blood parasites. Through the bite of an infective tsetse, people and animals may contract trypanosomiasis, a degenerative disease leading to death if left untreated. Tsetse control proved effective for disease containment, but data on the flies as tsetse identification are a prerequisite for planning any control intervention. There are 32 generally accepted tsetse species and subspecies. Classical species identification relies on minor morphological differences, often challenging for field workers. In the last decade, Matrix-Assisted Laser Desorption/Ionisation (MALDI) has revolutionised microbial species identification. After a simple protein extraction, a laser-induced ionisation takes place. Then, the ions are accelerated in a vacuum tube, and their Time of Flight (ToF) to reach the detector is recorded. The protein composition of each organism is unique, and so is their MALDI signature. Comparison of the obtained signature with a database of known organisms enables rapid identification as reliable as genome-based methods. To possibly speed up tsetse diagnostics, we established a MALDI database for the identification of five defined laboratory tsetse breeds. Inclusion of wild-caught tsetse could reinforce the reference database for the identification of tsetse at the species and subspecies level.

## Introduction

The trypanosomiasis infection risk of a particular area is determined by several factors, including tsetse species abundance and the sex distribution of a fly population [1]. While the sex is easily distinguishable with the bare eye, species identification can be challenging because there are 32 recognised tsetse species and subspecies [2]. Differentiation relies on morphological differences in colour, size and on minimal male genitalia variations [3]. Recent genome-based analyses revealed the subspecies status of seemingly uniform riverine *G. palpalis palpalis* individuals in Equatorial Guinea [4]. Accordingly, current tsetse specification based on morphology may not be the only way to rapidly determine the species status of *Glossina* spp.

The matrix assisted laser desorption/ionisation time of flight mass spectrometry (MALDI-TOF MS) is an established method of identification for microorganisms [5], [6], [7], [8], [9], [10], [11]. The MALDI-based identification of microorganisms requires only a small portion of a microbial colony and a drop of matrix solution [12], [13], [14]. The intact microbial cells are mixed with matrix solution (UV observing substances like alpha-Cyano-4-hydroxycinnamic acid, 2,5-dihydroxybenzoic acid), dried and subjected to laser induced soft ionization. The ions are then accelerated into a vacuum tube using a high electric field and the Time of Flight (ToF) to reach the detector is recorded. The velocity of an ion is inversely proportional to its mass, thus smaller ions travel faster than heavier ones and ions with the same charge travel together. The ions hitting the detector and their time of flight are visualized as spectra. The protein composition of each organism is unique, so a species-specific MALDI signature or spectrum is expected. The species identification does not require protein sequence data; instead the acquired spectra are matched with reference spectra database using a pattern- matching algorithm [9], [11]. The technique proved to be time and cost effective, as reliable as genome-based identification methods [6], [9]. Recently, MALDI-based species identification has been demonstrated for higher organisms as micro-algae, *Prototheca* [15], [16], the plant parasitic nematode *Anguina tritici* [17], *Drosophila* [18], [19], ticks [20] biting midges (*Culicoides* spp.) [21], [22], [23] and fish [24]. In addition MALDI has also been utilised for differentiation of various eukaryotic cell lines [25], immune cells [26], [27] and for species level classification of ancient mammalian samples [28].

Several commercial software packages designed for microbial species identification are available and include, MALDI Biotyper (Bruker Daltonics), the Axima (Shimadzu)-SARAMIS (AnagnosTec) systems (now called VITEK MS) (BioMérieux), Andromas (Andromas SAS) systems and MicrobeLynx (Waters) [7], [8], [29]. As far as our knowledge is concerned, reference spectra data for insects or tsetse in particular have not been included in any of these software packages. We chose the MALDI Biotyper system for creating a tsetse-specific spectra database. This system calculates the log score value, or similarity score, by considering the matching proportion of the test spectra with the database reference spectra. It also considers the consistency of peak intensities among sample and reference spectra.

The objective of this study was to investigate whether simple formic acid/acetonitrile extracts of five well known laboratory-reared tsetse breeds exhibit specific and reproducible peak patterns and if they prove to be valid for species level identification. Usually, field-collected tsetse are stored in ethanol and often parts of the insects are removed for diagnostics. Therefore, another goal was to investigate if any of the body parts (head, thorax, abdomen, legs, wings and whole insects) are useful for species prediction.

## Materials and Methods

### Tsetse selection and storage

To establish a tsetse database, we utilised five well-established laboratory breeds listed in table 1. They represent tsetse from three different habitats that are relevant for the transmission of trypanosomes that affect humans or animals [2]. Tsetse puparia were maintained at 26°C with a relative humidity of 75%. Two to 4 days after hatching they were sacrificed as tenerals at −18°C and then stored in ethanol (70%).

**Table 1:** Laboratory-reared *Glossina (G.)* spp. selected for the compilation of spectra database.

Species	Group	Tsetse Colony	Origin
*G. morsitans morsitans*	*Morsitans*	TTRI^1^	Kariba, Zimbabwe [37]
*G. austeni*	*Morsitans*	TTRI^1^	Zanzibar, Tanzania [38]
*G. pallidipes*	*Morsitans*	IAEA^2^	Tororo, Uganda [39]
*G. palpalis gambiensis*	*Palpalis*	IAEA^2^	Burkina Faso [40]
*G. brevipalpis* (red eye)	*Fusca*	IAEA^2^	Shimba Hills, Kenya [41]

1 Tsetse & Trypanosomiasis Research Institute, Tanga, Tanzania;

2 International Atomic Energy Agency, Seibersdorf, Austria.

### Fly dissection, protein extraction and MALDI measurement

A total of three insects each were obtained for the analysis of male and female entire individuals (table 1). Additionally, three males and females of each species were dissected representing the peak patterns of the body parts abdomen, head, legs, thorax and wings. The protein extraction was carried out as described in Murugaiyan et al. [16]. In brief, triplicates of each specimen (whole insect, head, thorax, abdomen, wings and legs) were washed with ethanol, air dried and mixed with equal volumes of 70% formic acid and 100% acetonitrile. The samples were then sonicated for 1 min on ice and the supernatants were collected for further analysis. One µl of each sample extract was spotted on to the MALDI target plate (MSP 96 target polished steel (MicroScout Target) plate Bruker Daltonics, Bremen, Germany), dried and overlaid with 1.0 µl of saturated α-cyano-4-hydroxycinnamic acid matrix solution. The MALDI measurements were carried out using MALDI Microflex LT (Bruker Daltonics, Bremen, Germany) on a broad range of 2000–20000 m/z (mass to charge ratio), following an external calibration with the bacterial test standard as recommended by the manufacturer. Each extract was spotted three times and each spot on the target plate was measured three times for acquiring 27 spectra per specimen. The spectra were acquired using the automated option (AutoXecute acquisition mode) in Flex control 3.0 software (Bruker Daltonics, Leipzig, Germany). (Box 1)

**Box 1. Key Steps in Maldi Microbial Identification and the Software Used in This Study**

With the aim of creating a simple protein extraction and identification procedure for tsetse, we utilized the well-established microbial method of MALDI identification:

1. Protein extraction
2. Spot on target plate, overlaid with matrix solution and dried
3. Spectra acquisition
4. Peak picking and pattern matching with the database

The protein extraction is an essential step for creating reference spectra of multicellular organisms while direct transfer of microbial colonies from the culture plate is sufficient for microbial identification. In this study commercial software associated with MALDI MicroFlex LT (Bruker Daltonics, Bremen, Germany) are utilized to create a tsetse specific database that draws from individual mass spectrum peaks. Spectra acquisition is carried out by the software Flexcontrol 3.0 and selection is performed manually after visualisation using FlexAnalysis 3.0. software. Final tsetse reference spectra were created with the software Biotyper 3.0 that includes the manufacturer's reference database.

### Sodium dodecyl sulfate polyacrylamide gel electrophoresis (SDS-PAGE)

In order to demonstrate the protein composition in each extract, *Glossina (G.) palpalis gambiensis* were chosen for an SDS-PAGE analysis [30]. In brief, the extracts of the whole insects and it's the body parts were precipitated in five volumes of ice-cold 100% acetone. The pellets were reconstituted with 10 µl of sample loading buffer, heated at 60°C for 5 minutes and separated using 4% stacking and 12% separating gel. The protein visualisation was carried out using Coomassie Blue staining [31].

### Data analysis and creation of tsetse reference spectra

Following the visual inspection using Flex analysis 3.0 software (Bruker Daltonics, Bremen, Germany), the spectra were then loaded in Biotyper 3.0 (Bruker Daltonics, Bremen, Germany) software. The spectra were subjected to baseline subtraction (multipolygonal; signal to noise ratio 3) and smoothing (Savitzky Golay algorithm, frame size 25 Da). The composite correlation index [32], a mathematical algorithm used to assess the spectra variations within and between each set of the measurements. The Composite Correlation Index (CCI) was computed using the standard settings of mass range 3000–12000 Da, resolution 4, four intervals and autocorrelation off. The reference spectra were then created using the standard method version 1.2 settings of the software (mass error of each single spectra: 2000, desired mass error of main spectra: 200, peak frequency: 25% and desired peak number: 70). The cluster analysis (main spectra dendrogram) was calculated with “correlation” as distance measure and linkage at “complete” to evaluate the suitability of the MALDI-based differentiation of tsetse at the species level. The created main spectra were then compiled as a tsetse database.

### Evaluation of the tsetse database

In order to check the suitability of the created tsetse main spectra for Biotyper-based species identification, the cross-matching status was created after matching them to the entire database. In addition, fresh extractions of the whole insect and the various insect parts were utilized in triplicates to cross-check the efficiency of the established tsetse database. For ruling out possible cross-matching with other fly species, the common house fly *Musca domestica* (Diptera: Muscidae; strain Lei) was also included in the evaluation. Identification was carried out using the Biotyper 3.0 software tool, following the manufacturer's recommendation on identification based on the calculated log score values. Values of ≥2.0 to 3.0 represent probable species level matching, while scores of ≥1.7 to 1.9 represent probable genus level matching. A score value of <1.7 stands for an unreliable identification.

## Results

**Figure 1:**
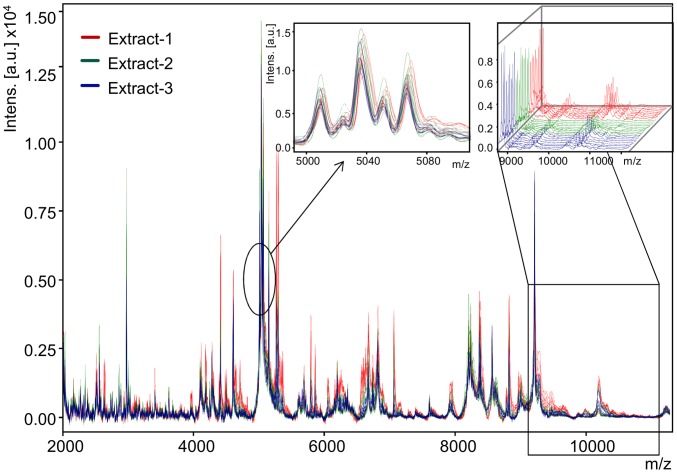
Spectra reproducibility among the biological and technical replicates. Overlay view of 27 spectra obtained from biological and technical replicates of *Glossina austeni* female whole insect. The masses (in Da) of the ions are shown on the *x*-axis and the *m/z* value stands for mass to charge ratio. On the y-axis, the relative intensity of the ions (a.u., arbitrary units) is shown. In the insert, zoomed m/z 5000 to 5200 displays the uniformity among the measured spectra and the stacked view m/z 9000 to 12500 provides a direct comparison of all 27 measured spectra.

From each tsetse specimen a total of 27 spectra representing biological and technical replicates in the m/z range of 2000–20000 Da were acquired automatically and thus 1620 spectra from whole *Glossina* species and their body parts A–J. Visual inspection of the spectra revealed a comparable peak pattern of the biological and technical replicates; however, differences in peak intensities were observed for example as shown in figure 1.

**Figure 2:**
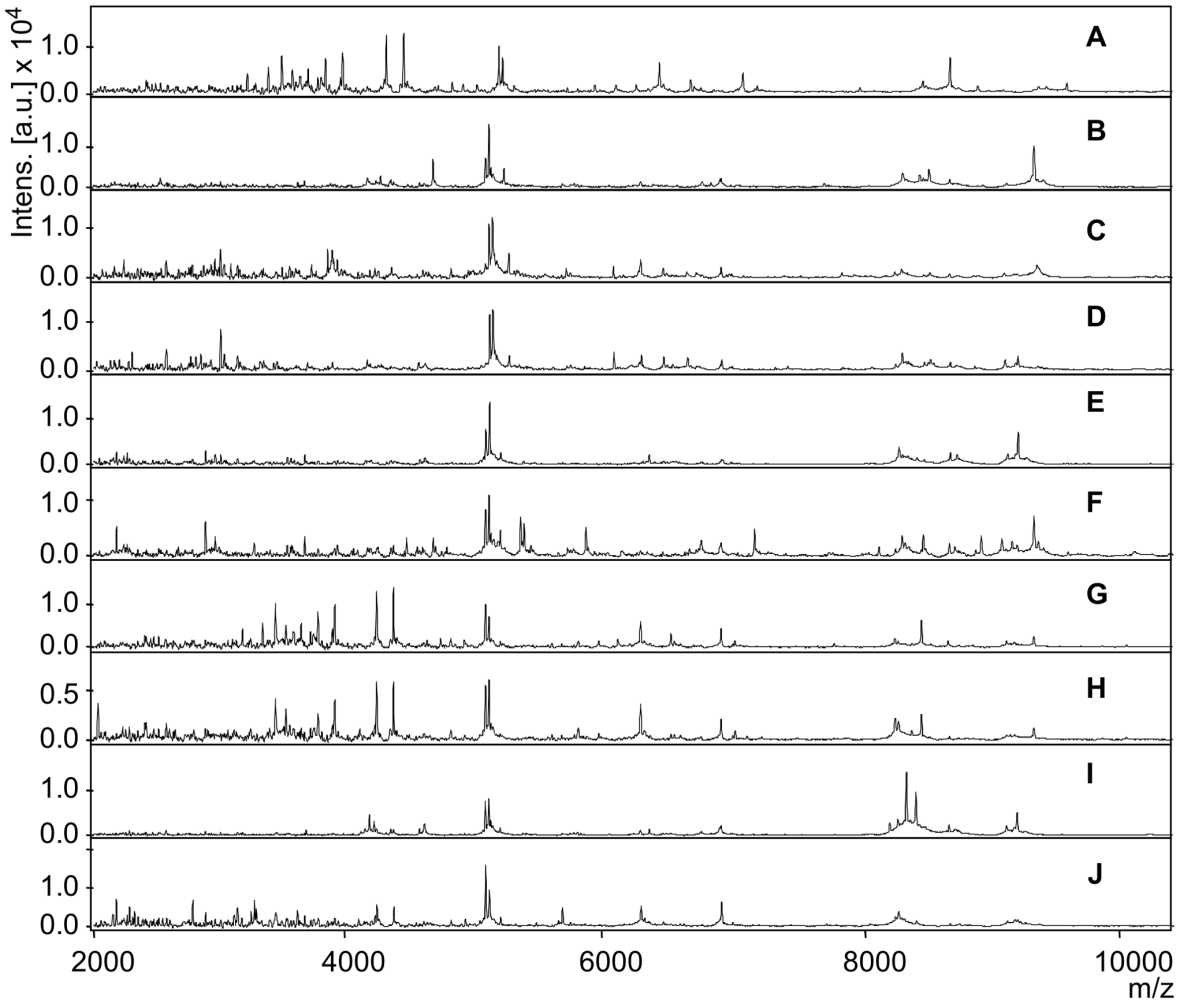
Representative spectra of whole insect extraction of male and female *Glossina* spp. Mass spectra peak pattern of whole insect extractions of male and female *Glossina (G.)* spp. The x-axis *m/z* value stands for mass to charge ratio and the relative intensity of the ions (a.u., arbitrary units) is shown on the y-axis. A) *G. morsitans morsitans* female, B) *G. morsitans morsitans* male, C) *G. austeni* female, D) *G. austeni* male, E) *G. pallidipes* female F) *G. pallidipes* male, G) *G. palpalis gambiensis* female, H) *G. palpalis gambiensis* male, I) *G. brevipalpis* female, and J) *G. brevipalpis* male.

At first look, the raw spectrum displayed consistently distinct peak patterns when comparing the two sexes of *G. palpalis gambiensis* (figure 2, samples G/H at m/z 5700, 7000 and 8000) while the three savannah species (A–F) and *G. brevipalpis* (I/J) only displayed differences in peak intensity. Occasionally observed differences as seen in the *G. pallidipes* female (sample E at 8100 m/z) appeared inconsistently. However, several peaks showed to be common for *Glossina* spp. as for instance presented in figure 2 at 5000 m/z.

**Figure 3:**
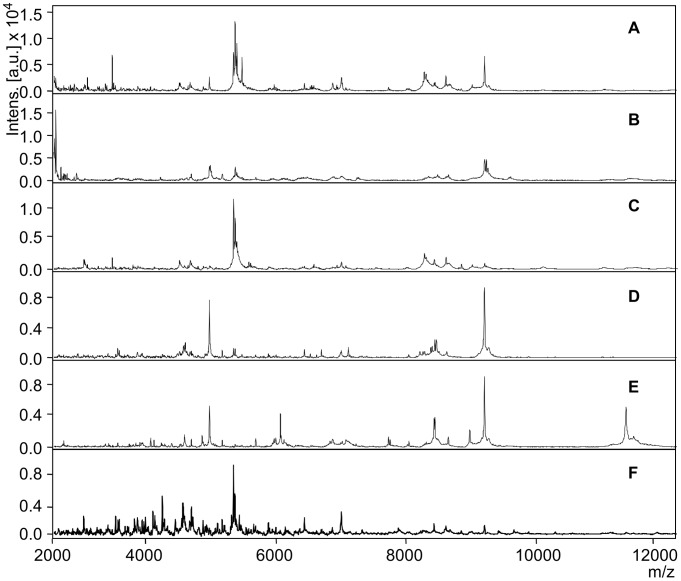
Representative spectra from the whole insect and different body parts of female *Glossina austeni*. Peak pattern of whole and body parts extractions of *Glossina austeni* female. The x-axis *m/z* values represent the mass to charge ratio and on the y-axis the relative intensity of the ions (a.u., arbitrary units) is shown. A) Whole insect, B) abdomen, C) head, D) legs, E) thorax and F) wings.

**Figure 4:**
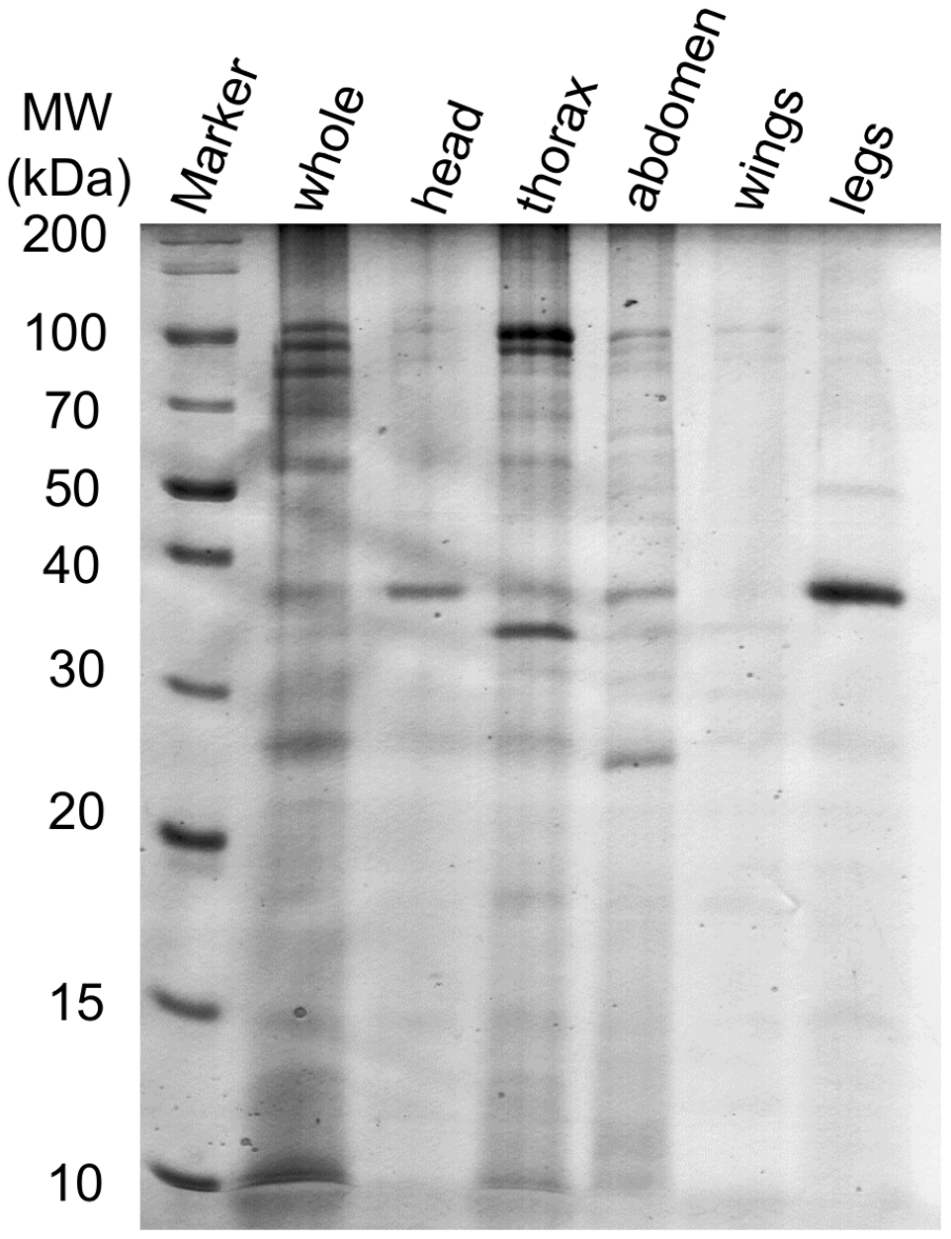
SDS PAGE separation of *Glossina palpalis gambiensis* protein extracts. SDS-PAGE separation of the whole insect and bodyparts of *Glossina palpalis gambiensis* extracts. 10 µg of proteins were run on 12% SDS-PAGE and stained with Coomassie brilliant blue.

As shown in figure 3, the raw spectra of different body parts and the entire insects presented varying peak patterns at least in terms of peak intensities. Among the body parts, peak intensities sometimes tended to be lower in some of the leg extracts when compared to entire insects or other parts. To demonstrate the protein composition of whole insects and the different body parts, *G. palpalis gambiensis* extracts were chosen for protein separation on SDS-PAGE and visualised using a modified Coomassie staining. As shown in figure 4, the protein separation was carried out from 10 to 200 kDa. The bands out of the extracts of the dissected body parts were clearly observed in the whole insect protein extract lane. However, it should be noted that the peaks in the MALDI spectra were obtained from much smaller peptides (2–20 kDa).

**Figure 5:**
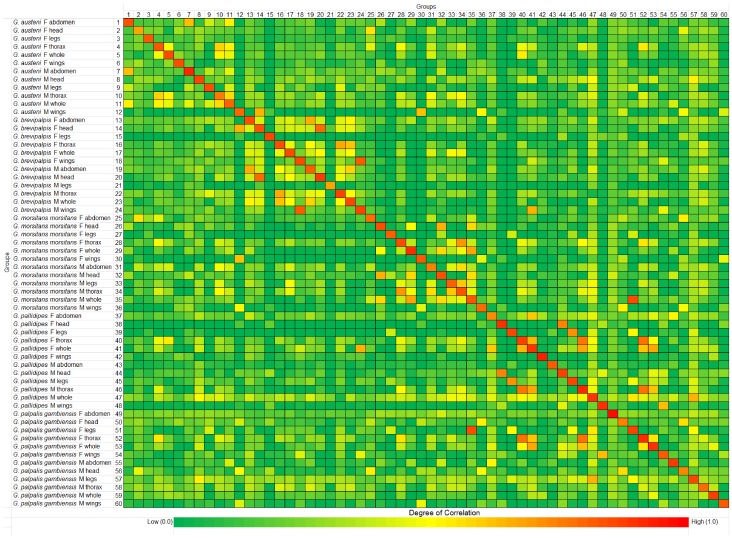
Composite correlation index of tsetse spectra sets. Evaluation of uniqueness among the spectra sets of 60 tsetse spectra measurements of male (M) and female (F) individuals and their body parts. Composite correlation index matrix was calculated with Biotyper 3.0 software in the mass range of 3000–12000 Da, resolution 4, 4 intervals and auto-correction off. Red indicates relatedness between the spectra sets and dark green indicates incongruence.

Figure 5 depicts the colour-coded computed composite correlation index [31] displaying the uniqueness of the acquired spectra 1–60. A CCI value of 0.0 (dark green) represents incongruency and 1.0 (red) denotes complete congruency. The CCI was observed between 0.68 and 0.98 (individual CCI values are shown in the supplement data table S1). Very few of the spectra sets displayed some deviation among themselves, for e.g. the CCI for *G. austeni* male head was 0.68. However, this spectra set displayed a complete deviation with other body parts or other species. Despite this shortcoming, the spectra sets appeared to be suitable for the compilation of a reference spectra library.

Cross–comparison of the tsetse main spectra with the entire Bruker reference database resulted in only one clear match with a log score value of >2.3, the cut-off value representing the most probable matching at the species-level. Some isolates such as *G. austeni* female head (no. 2) appeared to resemble *G. palpalis gambiensis* male head spectra (no. 56), however, the score value was distinctly lower than the expected matching set. This clearly indicated that these spectra sets could be utilized to establish a database.

**Figure 6:**
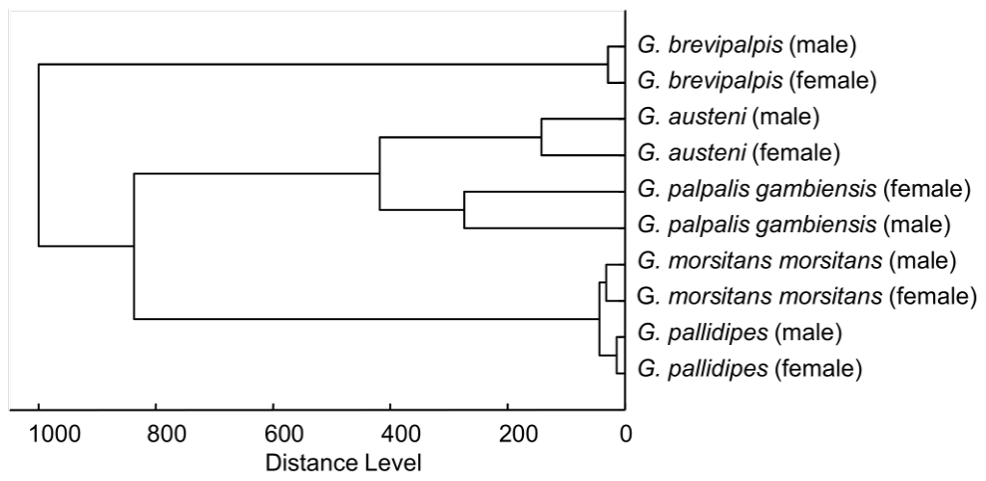
Score-oriented main spectra dendrogram of whole *Glossina spp*. extracts. The dendrogram was calculated by Biotyper 3.0 software with distance measure set at correlation and linkage set at complete.

Following these preliminary investigations, the main spectra library representing the 70 most reproducible peaks was constructed. The cluster analysis of the 10 main spectra of each species is shown in figure 6 for both sexes. Consistent clustering was observed among the extracts of *G. brevipalpis*, which always stood out as a sister group to the other species regardless of the body part. Furthermore, *G. austeni* showed inconsistent clustering, neither similar to savannah group tsetse nor to riverine *G. palpalis gambiensis* as for instance seen in the dendrogram.

The created tsetse main spectra were incorporated into the commercial Bruker system and then compared with the whole database following the manufacturer's recommendation. Accordingly, table S2 of the supplementary data describes the matching of tsetse main spectra where log score value 3.0 stands for a 100% match and lower matching probabilities were displayed as subsequent hits. The results indicate that the second hit within the acceptable cut-off value of >2.0 for some of the body part extracts matched with the correct body part but irrespective of the factors sex and species. This cross matching of body parts was predominantly observed between *G. austeni* and *G. morsitans morsitans* and among *G. pallidipes* and *G. palpalis gambiensis.* Within the same species, complete deviation was observed in *G. austeni* female head with its own abdomen and legs, Similarly, *G. palpalis gambiensis* female head did not match with its legs and thorax. *G. palpalis gambiensis* male head also displayed complete deviation with *G. palpalis gambiensis* female head.

As shown in table 2 (detailed identification results are listed in supplementary table S3), the results of fresh sample identification clearly indicate that every body part and sex was correctly matched at the species level (log score value >2.0). Despite the 100% correct identification, within this high confident identification the following score inconsistencies occurred: 58% (35/60) matched with the correct body part but also with the ones of the opposite sex, 35% (21/60) matched with the correct sex but with different body parts, 16% (10/60) matched with a different body part and the opposite sex and 5% (3/60) even matched with other species. The second best matching hits indicate that about 23% (14/60) of body parts displayed lower cut-off values (log score <1.7). Among the second best hits, incorrect matching was observed among 13 samples (21%): body parts of female *G. palpalis gambiensis* (thorax, whole and abdomen), *G. pallidipes* (female thorax) and *G.austeni* (male legs). The extracts from *Musca domestica* resulted in no reliable identification.

**Table 2:** Matching of five lab-reared tsetse with the Bruker reference database.

Log score value*	Interpretation	Correct Species				Incorrect species
		Correct sex and body part	Correct body part but incorrect sex	Correct sex but incorrect body part	Incorrect sex and body part	
2.30–3.00	Highly probable species	44	12	4	1	3
2.00–2.29	Probable species	16	23	17	9	16
1.70–1.99	Probable genus	0	11	14	20	21
0.00–1.69	Not reliable	0	14	25	30	20

* The manufacturer's recommended cut-off values were utilised to interpret the results.

## Discussion

To establish a tsetse reference database five laboratory breeds representing epidemiologically important tsetse of the savannah type *G. morsitans morsitans*, *G. pallidipes and G. austeni*, a riverine type *G. palpalis gambiensis* and forest type *G. brevipalpis* were chosen for this study [2], [33], [34] . Earlier attempts on the identification of arthropods by MALDI were carried out after homogenisation of the samples and extraction in a mass spectrometry-compatible buffer system [18], [20], [21]. We used a standard formic acid/acetonitrile extraction procedure of microbial cell processing for the protein extraction from tsetse. We introduced an additional step of sonication in order to facilitate the breakage of the chitin shell for a better protein yield. This simple extraction method was chosen to accommodate the field-collected samples that are stored in ethanol and possibly dissected.

Flex analysis software revealed that the spectra of the same species appeared to be fairly comparable despite the varying peak intensities. Visual inspection of the spectra revealed differences among the body parts of the same insect. Often, the most intense peaks of body part extracts were not easily observable in the spectra of whole insect extracts. This could be due to the protein ionisation influenced by varying protein compositions/abundances of different body part extracts. Additional evaluation of the protein composition/abundance using SDS-PAGE protein separation revealed the difference in protein bands. However, the bands of the body part extracts were comparable to those of the whole insect but they varied in their intensities. This was also shown among the different sexes of the same species. As the protein separation was carried out in a higher range (10 to 200 kDa) but the MALDI spectra stemmed from a much smaller range of proteins (2 to 20 kDa), So, a direct correlation among these could not be expected. However, the compositional protein differences among the various body part extracts and the whole insect are clear. This protein compositional difference might attribute to the observed difference among the spectra from different insect body parts. Despite this variation, the technical and biological replicates appeared to influence the peak intensity while the peak pattern was almost comparable.

Among the commercially available software tools for species identification, we used Biotyper software that incorporates 4613 main reference spectra of microbial species (March 2013). The software automatically pre-process the spectra through smoothing and baseline subtraction. The peaks were picked and compared with the reference database. The results were expressed as similarity log score values between 3.0 (complete matching) to 0 (complete deviation). As a first step of the main spectra creation, the practical relation among the spectra sets was visualised by computing the composite correlation index [32]. A CCI value approaching 1 is considered to be highly significant while zero represents complete deviation. A clear distinction between the spectra sets of different body parts and the whole insect extracts was displayed in the heat-map and its corresponding value. Some of the spectra set displayed signs of deviation, for e.g. *G. austeni* male head. This might be due to the presence of broader peaks, which did not overlap with the corresponding spectra [32]. The heat-map and CCI values indicated that the spectra sets of different body parts and the whole insect extracts were unique and could be utilised for the creation of a spectra library. Therefore, we generated 60 main spectra for five tsetse species including male and female whole insect extracts and the corresponding body parts. These main spectra were then incorporated in the Bruker database.

The main spectra dendrogram was useful for the differentiation of the five species, picturing the similarities and differences of their mass spectra profiles. Clustering of the created tsetse main spectra revealed that they did not follow any distinct pattern with some significant exceptions. A possible explanation could be that higher organisms like insects might not cluster at the species level using MALDI measurements unless they are being standardised. However, *G. austeni* never clustered clearly with riverine nor savannah species; it seems to share mass spectra patterns with both groups reflecting the uncertainty of their phylogenetic status [35]. Very clearly though was the uniqueness of *G. brevipalpis* compared to the other species. The sister status deriving from genomic findings [36] could therefore be mirrored in the mass spectrum peaks of *G. brevipalpis*.

As a quality check, tsetse main spectra were cross-identified with the entire database from the manufacturer. All the tsetse main spectra matched with a log score of 3.0, indicating a clear distinction between the species. It also showed the uniqueness of the tsetse mass spectra for entire tsetse as well as every dissected body part. Among the second best matched hits, sex and species appeared to be least important while the body parts across the species matched, especially among *G. austeni* and *G. morsitans morsitans* and also in *G. pallidipes* and *G. palpalis gambiensis* extracts. The complete deviation of head extracts (*G. austeni* female, *G. palpalis gambiensis* female and male) indicates special attention when working on species identification of head samples by MALDI.

The fresh protein extracts using the same insects resulted in 100% matches with the database. No hits were achieved for similarly processed *Musca domestica* extracts, indicating the uniqueness of the created reference spectra for tsetse. Among the best hits at the species level, body parts of the same species appeared to be matched correctly but irrespective of the sex. A deviating species in the second hit might be due to the presence of shared metabolic proteins among different tsetse species. The 5% that mismatched completely and the incorrect matching among the second hits indicate that the reference database should be created for more than one body part and of both sexes for reliable identification of insects.

The overall results clearly indicate that the success in MALDI-based identification relies on the specific signature from the body parts and the whole insects. While the first hit for these lab breed tsetse appeared to be specific for species, sex and body parts, the second hit indicates that sex is the least reliable feature of MALDI identification. The complete deviation of head extracts with its own other body parts as seen among *G. austeni* and *G. palpalis gambiensis* indicate that more than one body part is needed for accurate species identification. We propose the addition of spectra from field-caught tsetse (whole insects and body parts) to extend our database for a fast and accurate identification of tsetse.

## Supporting Information

Table S1 **Composite Correlation Index (CCI) values of 60 spectra sets of tsetse.** CCI was calculated using Biotyper 3.0 software (Bruker Daltonics, Bremen, Germany) in the mass range of 3000–12000 Da, resolution 4, 4 intervals and auto-correction off. CCI value nearing 1.0 indicates the relatedness between the spectral set and 0 indicates deviation among the spectra sets. M-male and F-female.
(XLSX)

Table S2 **Cross matching values of tsetse main spectra.** The created tsetse main spectra were selected in Biotyper 3.0 (Bruker Daltonics, Bremen, Germany) software and matched with the entire database. The log score value 3.0 indicates complete matching and 0 represents complete deviation. The manufacturer's recommended log score values, ≥2.0 to 3.0, ≥1.7 to 1.9 and <1.7 were utilised to interpret the identification as probable species level, genus level and no reliable identification respectively.
(XLSX)

Table S3 **Identification results of freshly extracted tsetse samples.** The insect proteins from whole insects and its body parts were extracted using formic acid/acetonitrile. 1.0 µl of the extracted was spotted on the target plate, air dried, 1.0 µl of saturated HCCA matrix was overlaid and dried completely. The result interpretation was carried out in accordance to the manufacturer's recommended cutoff log score values for species (≥2.0 to 3.0), genus (≥1.7 to 1.9) and the value lesser than 1.69 indicated that the samples were not reliable matched with any of the reference spectra.
(XLSX)
